# Supplementary material for: Factors Associated with Dietary Patterns of Schoolchildren: A Systematic Review
Source: Nutrients. 2023 May 24;15(11):2450. doi: 10.3390/nu15112450 (PMC10255100; doi:10.3390/nu15112450)
Supplement: Supplementary file 1 [file nutrients-15-02450-s001.zip › nutrients-2386468-supplementary.pdf]

Table S1 - Search Strategies

BASE	STRATEGIES
BVS Portal	("Food Consumption" OR "Consumo de Alimentos" OR "Consumo Alimentar" OR "Padrão Alimentar" OR "Padrão de Consumo de Alimentos" OR "Dietary Patterns" OR "Food Intake Pattern") AND (child OR niño OR criança OR crianças OR "Child Nutrition" OR "Nutrición del Niño" OR "Nutrição da Criança" OR "Alimentação Infantil" OR "Alimentação da Criança") AND ("Fatores de Influência" OR "Influencing Factors" OR influência OR influence) AND (db:("LILACS" OR "IBECS" OR "BDENF" OR "BBO" OR "INDEXPSI" OR "BINACIS" OR "CUMED" OR "SES-SP" OR "colecionaSUS"))
Pubmed	("Food Consumption" OR "Dietary Patterns" OR "Food Intake Pattern") AND (Child OR "Child Nutrition") AND ("Influencing Factors" OR Influence)
Scopus	("Food Consumption" OR "Dietary Patterns" OR "Food Intake Pattern") AND (Child OR "Child Nutrition") AND ("Influencing Factors" OR Influence)
Web of Science	("Food Consumption" OR "Dietary Patterns" OR "Food Intake Pattern") AND (Child OR "Child Nutrition") AND ("Influencing Factors" OR Influence)
